# Validity and reliability study of the dental students of attitudes towards online learning scale

**DOI:** 10.1186/s12909-025-08296-z

**Published:** 2025-12-22

**Authors:** Kamber Kaşali̇, Didem Özkal Emi̇noğlu, Şifa Demi̇rer Üstündağ

**Affiliations:** 1 Faculty of Medicine, Department of Biostatistics, Atatürk University, Erzurum, Türkiye; 2 Faculty of Dentistry, Department of Periodontology, Atatürk University, Erzurum, 25000 Türkiye

**Keywords:** Online learning, Dental education, Validity and reliability, Attitude scale

## Abstract

**Background:**

The shift to online learning during the COVID-19 pandemic highlighted the need for reliable tools to evaluate students’ attitudes. This study aimed to adapt the “Medical School Students’ Attitudes Towards Online Learning Scale” for dental students and assess its validity and reliability.

**Methods:**

A cross-sectional study with 181 dental students was conducted. Data were analyzed for mean, standard deviation, count, and percentage. Validity analyses included the Kaiser-Meyer-Olkin (KMO) test and Bartlett’s Sphericity test for factor analysis suitability. Exploratory Factor Analysis (EFA) and Confirmatory Factor Analysis (CFA) assessed construct validity. Reliability was evaluated through Cronbach’s Alpha, item-total correlations, and split-half reliability. Model fit indices (χ²/df, RMSEA, CFI, TLI, SRMR) were used to ensure acceptable goodness-of-fit values. Analyses were conducted using SPSS 20.0 and JAMOVI 2.5.3, with *p* < 0.05.

**Results:**

The first-order CFA model aligned with the original structure (χ²/df = 2.336, RMSEA = 0.080, SRMR = 0.069, CFI = 0.893). Cronbach’s alpha was 0.938 for the entire scale and ranged from 0.876 to 0.930 for subdimensions. Item reliability analyses showed alpha values from 0.933 to 0.939, while split-half reliability coefficients ranged from 0.865 to 0.930. Hotelling’s T² test revealed significant differences in item means (*p* < 0.001).

**Conclusions:**

The adapted scale exhibited strong psychometric properties, making it a reliable tool for assessing dental students’ attitudes towards online learning.

**Supplementary Information:**

The online version contains supplementary material available at 10.1186/s12909-025-08296-z.

## Introduction

In today’s world, where technology is continuously advancing, the internet has become an integral part of our lives. The development of information and communication technologies has driven the restructuring of educational systems and institutions. In education, there has been a growing demand for learner-centered plans that can continue throughout life, regardless of time and place. Institutions have recognized the importance of transitioning to educational models that prioritize these variables, and have been developing their content programs accordingly [1].

One of the electronic learning methods is online learning. Online learning is a method where at least one of the three components—teacher, student, and course content—is located in a different space, and these educational elements are integrated using various technological applications to facilitate education. The success of e-learning depends on several factors, including accessibility, the use of appropriate methods, course content, and assessment criteria [2, 3].

Like other teaching methods, e-learning has both advantages and disadvantages for students and educators. The primary advantages of online learning include time savings, cost reduction, the provision of multimedia that accommodates different learning styles, enabling students to learn outside the classroom at any time, addressing teacher shortages, and the potential to shift the learning process from passive, teacher-centered education to active, student-centered learning [4–6]. However, disadvantages such as technology-related apprehension, time management difficulties, and occasional connectivity issues encountered during synchronous sessions should not be overlooked [7, 8]. With the increasing opportunities today, these disadvantages are gradually being eliminated. Most dental students at the undergraduate level are computer literate and supported by institutions with extensive information and technological infrastructure [9].

E-learning has gained popularity over the past decade, and it is more prevalent in basic science courses than in clinical internships [10, 11]. In other words, online learning is generally not used as a standalone method for the education of undergraduate students but is integrated with the traditional teaching plan (teacher-led approach). This form of education can be referred to as a blended or hybrid learning approach [12, 13].

The goal of dental education is to train clinicians who prioritize public oral and dental health. Dental education consists of three different components: theoretical courses, preclinical training, and clinical training [14]. A process can be described in dental education where practical and theoretical training are integrated, with practical training initially provided in the form of preclinical education in the early years, followed by clinical education in later stages. In the preclinical phase of dental education, psychomotor skills are developed on models and mannequins under the supervision of instructors [15, 16]. Clinical education, on the other hand, complements the preclinical training and is essential for acquiring the ability to treat patients independently. During this phase, students, as trainees, interact with patients and participate in their necessary treatments under the supervision of instructors at their clinical placements. Through this process, students acquire experience, competence, knowledge, problem-solving skills, and critical thinking abilities [15, 17]. Due to these reasons, while the adaptation of theoretical dental courses to distance education may be relatively simpler, the adaptation of practical courses, which are focused on professional skills and require one-on-one interaction, is less feasible [18]. Although efforts are made to develop alternative methods, face-to-face education remains the primary method for dental school courses focused on practical and clinical education. For this reason, before the COVID-19 pandemic, online education had not been fully adopted as a method of instruction in dental and medical faculties in Turkey or worldwide [19–21]. However, the pandemic period forced dental faculties, in particular, to transition to remote learning [18]. The COVID-19 pandemic has necessitated the use of current technologies in education [17] and has posed challenges for both students and educators regarding practical training [18]. During this period, issues such as anxiety about the future, concerns about inadequate education, depression, and anxiety were reported among students who could not receive in-person training [22, 23].

There are studies in the literature that examine the usage habits, attitudes, perceptions [20, 21], and readiness levels for e-learning [24] of undergraduate dental students in the online learning process. These studies have revealed that students believe online education cannot fully replace traditional education; however, they have noted that students view the continuation of education under pandemic conditions as a positive development. In addition, it has shown that students have expressed needs such as improving course design, developing teacher training programs, addressing the lack of interaction, and planning course durations appropriate for attention spans. Furthermore, it has highlighted concerns that e-learning may cause negative effects for students, such as isolation and screen fatigue.

While several studies have investigated dental students’ perceptions of online education, the majority have relied on ad hoc questionnaires or scales with limited psychometric validation, often focusing mainly on readiness or satisfaction rather than attitudes [25–29]. In contrast, the scale originally developed for medical students was selected because it provides a multidimensional measure of attitudes toward online learning with proven psychometric robustness, and it had undergone factorial validation. Our adaptation demonstrated excellent reliability (Cronbach’s α = 0.938) and acceptable fit indices (CFI = 0.893, RMSEA = 0.080), thereby ensuring methodological rigor and cross-cultural applicability.

This adaptation not only addresses the shortcomings of existing dental education instruments but also designed to add value by introducing the first validated, reliable, and comprehensive tool specifically for dental students’ attitudes toward online learning. Thus, the present study also substantially endeavors to contribute to the literature by providing a scale that is both psychometrically sound and directly relevant to dental education.

This study aims to adapt the “Online Learning Attitudes Scale for Medical Students,” developed by Mustafa Onur Yurdal et al. [30] for dental students and conduct validity and reliability analyses of the scale for dental students. Additionally, it seeks to examine whether the scale is an effective tool for evaluating dental students’ attitudes toward online learning.

## Materials and methods

### Study design

This study is a methodological scale adaptation study designed as a validity and reliability analysis. In the initial phase, permission was obtained from the authors who developed the original scale to adapt it for dental students. Ethical approval was obtained from the Atatürk University Faculty of Dentistry Ethics Committee (meeting date: 28/03/2024; meeting number: 3; approval number: 37). The study was conducted in accordance with the ethical standards of the institutional and national research committee and with the 1964 Declaration of Helsinki and its later amendments.

### Setting and participants

The population of the study consisted of students enrolled in the Faculty of Dentistry at Atatürk University between 30.07.2024–15.08.2024. Written informed consent to participate was obtained from all participants prior to data collection. Participation was voluntary, and all participants were informed about the purpose, scope, and confidentiality principles of the study. The study group was composed of 181 students selected through a purposive sampling method. The 30 students who participated in the pilot study were excluded from the main study. During the data collection process, participants were informed about the purpose, scope, and confidentiality principles of the study. Only students who provided voluntary consent were included in the study.

Data collection was carried out using a questionnaire, which did not request personal identification information, and the data were collected anonymously. The survey took approximately 17 min to complete, and participants were provided with a consent checkbox to confirm their agreement to participate in the study.

### Sample size

The sample size was calculated based on the Cronbach’s α value. To achieve a Cronbach’s α of 0.88 with a 95% confidence interval and 80% statistical power, it was determined that a minimum of 180 students needed to participate in the study. Considering the possibility of data loss, the study was conducted with 181 students.

### Data collection tools

For the purpose of data collection, the adapted version of the “Medical Students’ Attitudes Towards Online Learning Scale” for dental students and a demographic information form were utilized. Participation to the survey was voluntary, and there were no consequences or benefits associated with it. The questionnaire was distributed to students via the online survey platform “docs.google” [31]. Participant confidentiality was maintained with no names recorded on the questionnaire.

### Medical school students’ attitudes towards online learning scale

Developed by Yurdal et al. [30] based on a study involving 815 medical students, this scale was designed to measure medical students’ attitudes towards online learning. The scale comprises 22 items divided into two subdimensions: “Attitudes Towards Online Learning (ATOL)” and “Attitudes Towards Online Medical Education (ATOME).” The ATOL subdimension, consisting of 11 items, evaluates the general benefits and compatibility of online education, while the ATOME subdimension, also consisting of 11 items, assesses the specific effects of online learning on medical education. The scale is a 5-point Likert-type instrument, with responses ranging from “Strongly Disagree (1)” to “Strongly Agree (5).” It has demonstrated high reliability, with Cronbach’s Alpha coefficients of 0.97 for the total scale, 0.96 for the ATOL subdimension, and 0.92 for the ATOME subdimension. The total score ranges from 22 to 110, with higher scores indicating more positive attitudes towards online education.

Additionally, participants were asked demographic variables such as their age, gender, academic year, and reasons for choosing dentistry.

### Procedures conducted for scale adaptation

Following written permission from the original developers and ethics committee approval, the adaptation of the scale for dental students was carried out in accordance with international guidelines and recommendations [32–35]. A multi-step forward–backward translation and expert review process was implemented.

Forward translation and reconciliation: The original scale was independently translated into Turkish by two forward translators who were proficient in English (C1/C2 level) and had at least five years’ experience in health and dental terminology translation. The two versions were compared in a reconciliation meeting attended by both translators, a dental academic, an educational scientist, and a linguist. Semantic, conceptual, and measurement-context equivalence were discussed item by item. Discrepancies were resolved through consensus, with justifications documented for each change.

Independent back-translation and committee review: The reconciled Turkish draft was independently back-translated into English by two native-English translators unfamiliar with the original instrument. Back-translations were compared with the source version to detect any meaning shifts, narrowing or broadening of scope, and inaccuracies in technical terms. Necessary wording adjustments were made, and the bilingual committee approved the final Turkish draft.

Expert panel and content validity: A five-member expert panel (three educational scientists and two dental academics) was formed based on predefined criteria: doctoral/PhD qualifications, publications in measurement/evaluation or dental education, and at least five years of relevant academic experience. Experts rated each item on a 4-point Likert scale for content relevance, clarity/comprehensibility, and cultural appropriateness, and provided qualitative comments. Item-level Content Validity Index (I-CVI) and Scale-level CVI (S-CVI/Ave) were calculated. Items below the accepted threshold or receiving substantive qualitative critiques were revised accordingly.

Pilot testing (cognitive interviews): The pre-final version was pilot-tested with 30 dental students drawn purposively to include both pre-clinical (years 1–3) and clinical (years 4–5) levels. Short cognitive interviews explored item clarity, interpretive consistency, familiarity of terminology, and suitability of response options. Completion time was recorded. Feedback led to minor linguistic and formatting adjustments—such as substituting simpler terms for technical jargon, splitting long sentences, and contextualizing examples for dentistry—while preserving the number of items and scoring structure. Pilot data were not included in the psychometric analyses, which were performed on the main study sample.

Transparent reporting (Table 1): Revised Table 1 presents, for each item: (i) the original English wording, (ii) the final Turkish wording, (iii) the final English translation, Based on the feedback received, final revisions were made, and the scale was finalized.

**Table 1:** Adaptation audit trail for the online learning attitudes scale for dental students

Item	Original English	Final Turkish	Back Translation	Type of Modification	Reason for Change	Number of Experts Giving a Score of 3 or 4 (2 Experts) (I-CVI)
1	Online access to lecture notes about lessons/internships is ideal for doctor training.	Derslerle/stajlarla ilgili ders notlarına online erişim diş hekimi yetiştirmede idealdir.	Online access to lecture notes about lessons/internships is ideal for dentist training.	#	1	2 (1.00)
2	The goals of medical education can be achieved using distance education methods.	Uzaktan eğitim yöntemleriyle diş hekimliği eğitimi amacına ulaşabilir.	The goals of dental education can be achieved using distance education methods.	&	2	2 (1.00)
3	Doctor training can be made by distance education.	Uzaktan eğitimle diş hekimi yetiştirilebilir.	Dentist training can be made by distance education.	#	1	2 (1.00)
4	The skills that a doctor should have can be provided online.	Diş hekimi yetiştirmek için gerekli beceriler online olarak kazandırabilir.	The skills that a dentist should have can be provided online.	#	1	2 (1.00)
5	The attitudes that a doctor should have can be gained online.	Diş hekiminin sahip olması gereken tutumlar online olarak edinilebilir.	The attitudes that a dentist should have can be gained online.	#	1	2 (1.00)
6	The communication way of a doctor with his patient and patient’s relatives can be taught online.	Diş hekiminin, hasta ve yakınlarıyla nasıl iletişim kuracağı online olarak öğretilebilir.	The communication way of a dentist with his patient and patient’s relatives can be taught online.	#	1	2 (1.00)
7	I can use the information that I gained through distance education (for example, measuring blood pressure) as a skill when I face with a patient.	Uzaktan eğitimle kazandığım pratik bilgileri (örneğin protetik ölçü alımı) hastayla karşılaştığımda beceri olarak sergileyebilirim.	I can use the information that I gained through distance Education (e.g., prosthetic impression taking) as a skill when I face with a patient.	¥	3	2 (1.00)
8	Patient consultation can be taught online.	Bir hastanın nasıl muayene edileceği online olarak öğretilebilir.	Patient consultation can be taught online.	∞	4	2 (1.00)
9	The skills about giving bad news (such as notifying of a death) can be taught online.	Zor haber verme becerisi (örn. Bir dişin çekimi) online öğretilebilir.	The skills about giving bad news (such as notifying of a extraction of teeth) can be taught online.	¥	3	2 (1.00)
10	Emergency medicine practice can be taught online.	Acil tıp uygulamaları online öğretilebilir.	Emergency medicine practice can be taught online.	∞	4	2 (1.00)
11	The classroom lessons in medical education can be provided online without any loss.	Diş hekimliği eğitimindeki sınıf dersleri bir kayıp oluşturmadan online olarak verilebilir.	The classroom lessons in dental education can be provided online without any loss.	&	2	2 (1.00)
12	Making classroom lessons through the online system contributes to personalize my study program.	Sınıf derslerinin online sisteme geçirilmesi, ders çalışma programımı kişiselleştirmeme katkı sağlar.	Making classroom lessons through the online system contributes to personalize my study program.	∞	4	2 (1.00)
13	Online education contributes to the use of audio-visual materials.	Online eğitim görsel-işitsel ögelerin kullanımına katkı yapar.	Online education contributes to the use of audio-visual materials.	∞	4	2 (1.00)
14	Online education is better than classroom education which has physical limitations.	Online eğitim fiziksel kısıtlılıklara sahip sınıf eğitiminden üstündür.	Online education is better than classroom education which has physical limitations.	∞	4	2 (1.00)
15	I adapt easily to online education.	Online eğitime kolayca uyum sağlarım.	I adapt easily to online education.	∞	4	2 (1.00)
16	Online education allows me to use my time more efficiently compared to classroom lessons.	Online eğitim zamanını sınıf derslerinden daha verimli kullanmamı sağlar.	Online education allows me to use my time more efficiently compared to classroom lessons.	∞	4	2 (1.00)
17	I like being informed about lecture notes through the distance learning environment.	Uzaktan eğitim ortamındaki ders notlarından bilgi edinmekten hoşlanırım.	I like being informed about lecture notes through the distance learning environment.	∞	4	2 (1.00)
18	Online access to lecture notes about lessons/internships, makes me feel free.	Dersler/stajlarla ilgili ders notlarına online erişim bana kendimi özgür hissettirir.	Online access to lecture notes about lessons/internships, makes me feel free.	∞	4	2 (1.00)
19	Online education is very rich since it’s audio-visual interactive.	Online eğitim görsel, işitsel, etkileşimli olarak büyük zenginliktir.	Online education is very rich since it’s audio-visual interactive.	∞	4	2 (1.00)
20	I encourage my classmates to take online education.	Sınıf arkadaşlarımın online eğitim almaları için onları teşvik ederim.	I encourage my classmates to take online education.	∞	4	2 (1.00)
21	I support all efforts to extend distance education.	Uzaktan eğitimi yaygınlaştıracak her tür çabayı desteklerim.	I support all efforts to extend distance education.	∞	4	2 (1.00)
22	I enable doctor training through distance education if I’m the Health Minister.	Sağlık bakanı olsam uzaktan eğitimle hekim yetiştirilmesini sağlarım.	I enable dentist training through distance education if I’m the Health Minister.	#	1	2 (1.00)
S-CVI						1.00

# : Terminology adaptation (“doctor” → “dentist”)

1: To align terminology with the relevant professional context and ensure clarity by replacing the general term “doctor” with the specific term “dentist”

& : Terminology adaptation (“medical” → “dental”)

2: To align terminology with the relevant professional context and ensure clarity by replacing the general term “medical” with the specific term “dental”

¥ : Example contextualization

3: Added example relevant to dentistry; expert panel and pilot feedback

∞ : None – retained wording

4: Clear and equivalent

### Statistical analyses

The data in our study are presented as mean, standard deviation, count, and percentage. For validity analyses, the Kaiser-Meyer-Olkin (KMO) test and Bartlett’s Sphericity test were conducted to determine the suitability of the data for factor analysis. To assess the construct validity of the scale, both Exploratory Factor Analysis (EFA) and Confirmatory Factor Analysis (CFA) were performed. Reliability analyses involved calculating the Cronbach’s Alpha coefficient to evaluate the internal consistency of the scale. Additionally, reliability was further detailed through item-total correlations and split-half reliability analysis. Finally, to evaluate the model’s fit, indices such as χ²/df, RMSEA, CFI, TLI, and SRMR were utilized, ensuring that goodness-of-fit values fell within acceptable ranges. Data analyses were conducted using SPSS 20.0 and JAMOVI 2.5.3 software, with a significance level of *p* < 0.05 for all analyses.

## Results

The study included 181 dental students, with 26.5% male and 73.5% female participants. Additionally, the majority (38.1%) were fourth-year students, and 51.0% stated that they enrolled in the faculty of dentistry because their exam scores qualified them for admission. The socio-demographic characteristics of the students are presented in Table 2.

**Table 2:** Results of Socio-Demographic data

		*N*	% of *N*
Gender	Male	48	26.5%
	Female	133	73.5%
Year of Study	1 st Year	16	8.8%
	2nd Year	17	9.4%
	3rd Year	21	11.6%
	4th Year	69	38.1%
	5th Year	58	32.0%
Reason for Choosing Dentistry	Due to Exam Scores	93	51.00%
	It Was My Dream Profession	53	29.00%
	Because My Family Wanted It	9	5.00%
	Due to Its Good Social Status	19	10.00%
	Because It Is a Financially Secure Career	7	4.00%

**Fig. 1:**
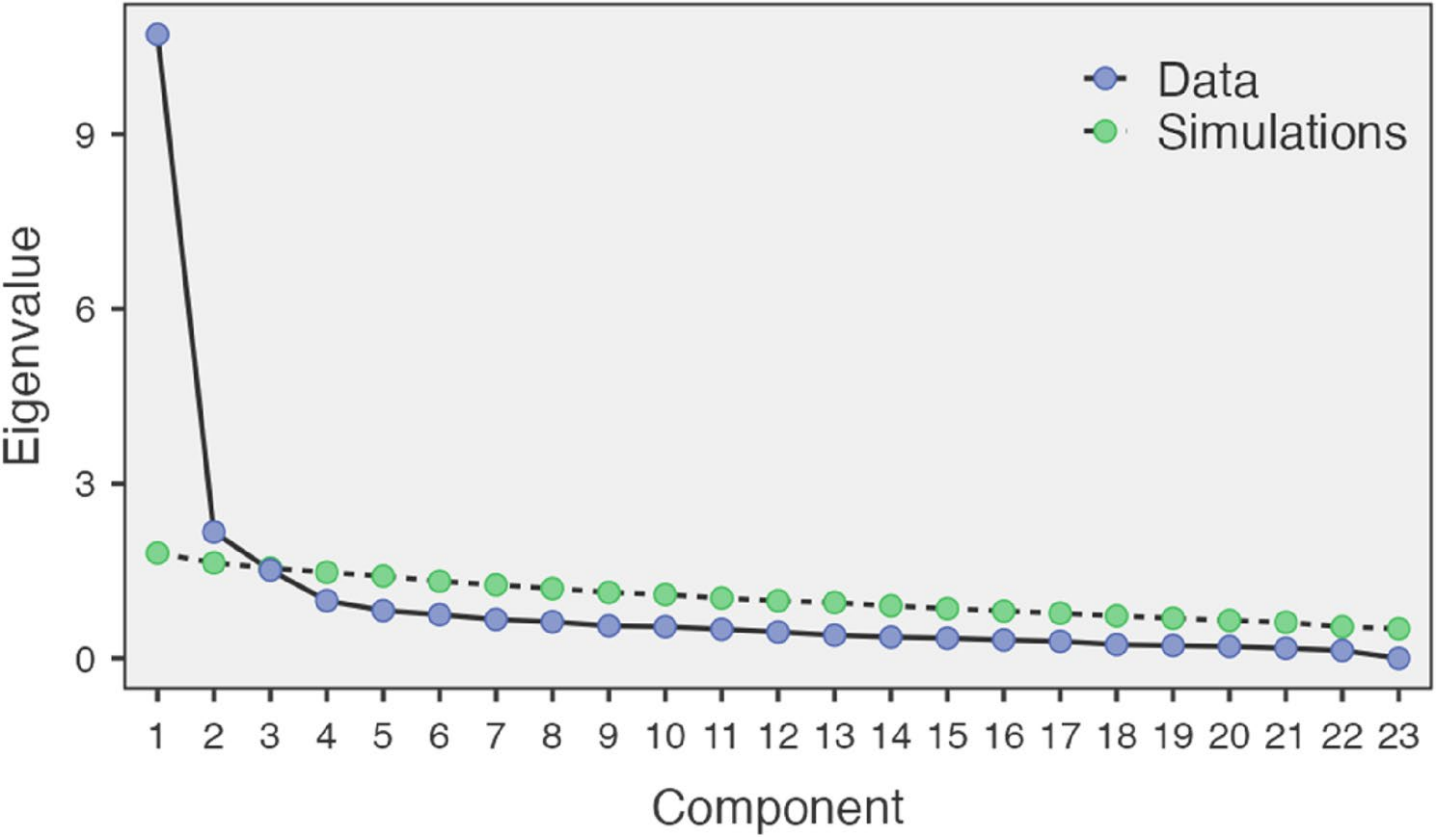
Scree Plot

Bartlett’s test of sphericity and the Kaiser-Meyer-Olkin (KMO) measure indicated that the scale was suitable for factor analysis. The KMO value was 0.867 for ATOL subdimension and 0.914 for (Attitudes Towards Online Dental Education) ATODE subdimension (ATOL > 0.8 is considered meritorious, and ATODE > 0.9 is excellent), with Bartlett’s test being statistically significant. The chi-square values were calculated as χ² = 881.0, degrees of freedom (DF) = 55, *p* < 0.001 for ATOL subdimension, and χ² = 1290.0, DF = 55, *p* < 0.001 for ATODE subdimension. Thus, it was determined that the “Online Learning Attitudes Scale for Dental Students” was appropriate for factor analysis. The scree plot revealed a clear elbow at the second component, confirming the two-factor structure of the scale. According to the scree plot test, the scale consisted of two components, with factors beyond the second being non-explanatory (Fig. 1; Table 3).

**Table 3:** KMO and bartlett’s test results

	KMO Measure of Sampling Adequacy	Bartlett’s Test of Sphericity		
		χ²	df	*p*
ATOL Subdimension	0.867	881	55	< 0.001
ATODE Subdimension	0.914	1290	55	< 0.001

*KMO* Kaiser–Meyer–Olkin, *χ²* Chi-Square, *df* Degree of Freedom, *ATOL subdimension* Attitudes Towards Online Learning, *ATODE subdimension* Attitudes Towards Online Dental Education

### Confirmatory factor analysis

**Fig. 2:**
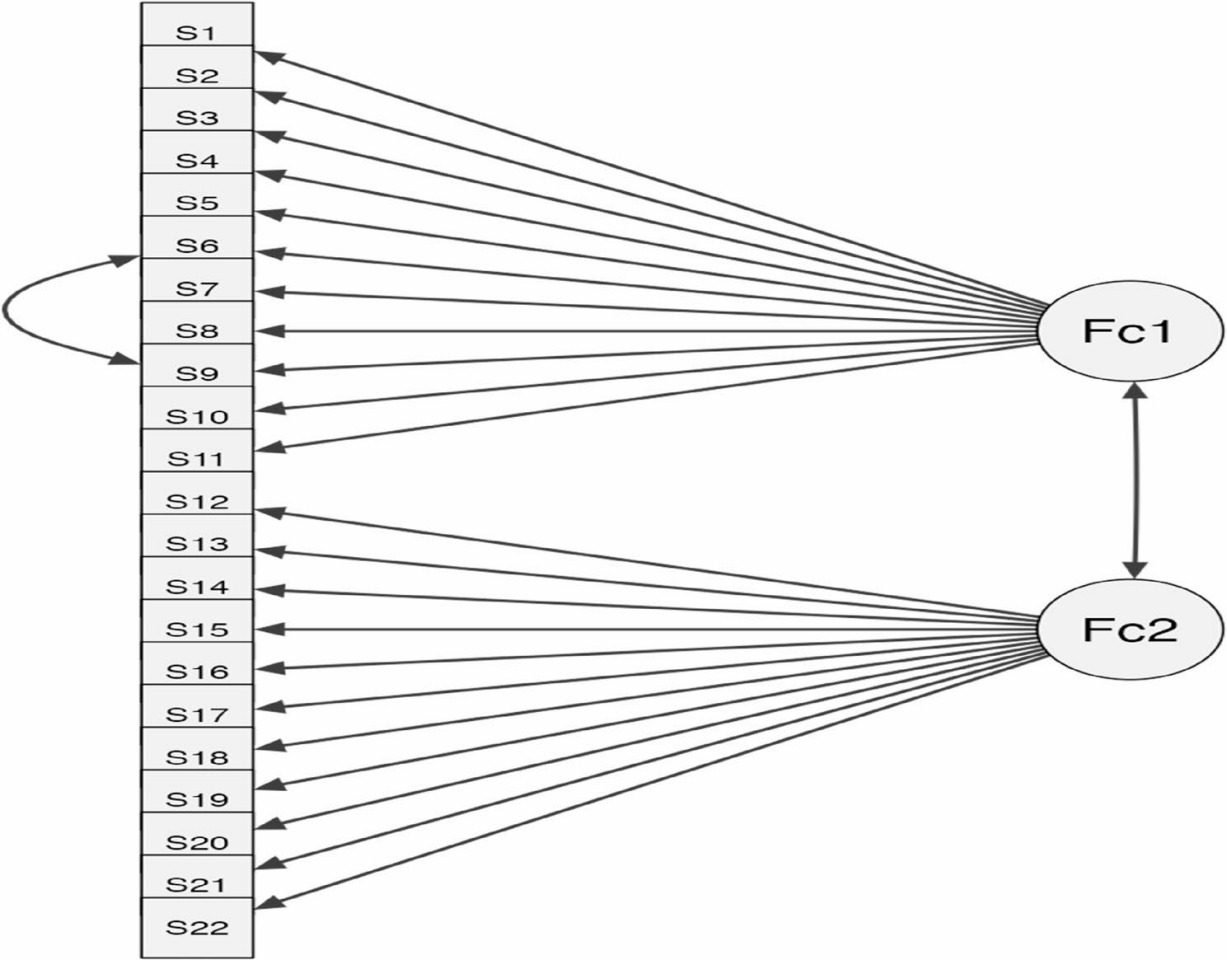
Path Diagram. Fc1: ATOL (Attitudes Towards Online Learning); Fc2: ATODE (Attitudes Towards Online Dental Education)

According to the first-order CFA model, 11 items (Items 1, 2, 3, 4, 5, 6, 7, 8, 9, 10, and 11) were grouped under the “Students’ Attitudes Towards Online Learning” subdimension, while another 11 items (Items 12, 13, 14, 15, 16, 17, 18, 19, 20, 21, and 22) were grouped under the “Students’ Attitudes Towards Online Dental Education” subdimension (Fig. 2). Table 4 demonstrates that each factor contributes to the model significantly (*p* < 0.05).

**Table 4:** Factor loadings results

Factor Loadings	Indicator	Estimate	SE	95% CI Lower	95% CI Upper	Z	*p*	Stand. Estimate
ATOL “Students’ Attitudes Towards Online Learning”	S1	0.725	0.0915	0.546	0.904	7.92	< 0.001	0.568
	S2	0.712	0.0659	0.583	0.841	10.8	< 0.001	0.724
	S3	0.557	0.0598	0.44	0.674	9.32	< 0.001	0.649
	S4	0.442	0.0483	0.348	0.537	9.16	< 0.001	0.641
	S5	0.841	0.0709	0.702	0.98	11.87	< 0.001	0.774
	S6	0.663	0.0854	0.496	0.831	7.76	< 0.001	0.563
	S7	0.507	0.0679	0.374	0.64	7.46	< 0.001	0.542
	S8	0.56	0.0636	0.436	0.685	8.81	< 0.001	0.619
	S9	0.677	0.0685	0.543	0.811	9.89	< 0.001	0.68
	S10	0.427	0.0665	0.297	0.558	6.42	< 0.001	0.475
	S11	0.883	0.0854	0.715	1.05	10.33	< 0.001	0.705
ATODE “Students’ Attitudes Towards Online Dental Education”	S12	0.977	0.0737	0.833	1.122	13.26	< 0.001	0.821
	S13	0.886	0.0781	0.732	1.039	11.34	< 0.001	0.738
	S14	0.929	0.0792	0.774	1.084	11.72	< 0.001	0.755
	S15	0.92	0.0781	0.767	1.073	11.79	< 0.001	0.759
	S16	1.045	0.0812	0.886	1.204	12.87	< 0.001	0.806
	S17	1.009	0.0794	0.853	1.164	12.7	< 0.001	0.798
	S18	0.718	0.0776	0.566	0.87	9.25	< 0.001	0.634
	S19	0.899	0.076	0.75	1.048	11.82	< 0.001	0.76
	S20	0.767	0.0667	0.636	0.898	11.5	< 0.001	0.755
	S21	0.848	0.0689	0.713	0.983	12.32	< 0.001	0.783
	S22	0.434	0.0583	0.32	0.548	7.45	< 0.001	0.532

*SE* Standard Error, *CI* Confidence Interval, *Z* Statistic Value

### Model fit of the scale

According to the goodness-of-fit analysis of the first-order CFA model, the model aligns with the original structure of the study (χ²/df = 2.336, RMSEA = 0.080, SRMR = 0.069, CFI = 0.893). Our analysis indicates that the χ²/df ratio of 2.336 indicates a good fit, while the RMSEA (0.080) and SRMR (0.069) values suggest acceptable fit. The CFI value of 0.893, though slightly below the threshold of 0.90, still indicates a reasonable fit for the model. The results of the fit analysis are presented in Table 5, alongside the reference values for commonly used goodness-of-fit indices in the literature.

**Table 5:** Results of model fit indices

Fit Index	Reference Value (Good Fit)	Reference Value (Acceptable Fit)	Measured Value	Result
CMIN/DF	0 < χ 2/SD ≤ 3	3 < χ 2/SD ≤ 5	2,336	Good Fit
RMSEA	0 ≤ RMSEA ≤,05	,05 < RMSEA ≤,08	0,080	Acceptable Fit
SRMR	0 ≤ SRMR ≤,05	,05 < SRMR ≤,10	0,069	Acceptable Fit
CFI	,95 < CFI ≤ 1	,90 < CFI ≤,94	0,893	Slightly below the acceptable threshold of 0.90.

### Findings on the reliability of the scale

In our study, the Cronbach’s alpha reliability coefficient was calculated as 0.938 for the entire scale, and 0.876 and 0.930 for the ATOL subdimension and ATODE subdimension, respectively. These values indicate high internal consistency, that the sub-items of the scale are consistent with each other and measure the same construct (Table 6). According to the analysis results for the reliability of the scale items, Cronbach’s alpha values ranged between 0.933 and 0.939. Item-total correlations ranged between 0.406 and 0.776, indicating that all items contributed adequately to the scale (Table 7). In the split-half reliability analysis conducted to evaluate the scale’s reliability, Cronbach’s alpha values were found to be 0.865 and 0.930. These results indicate that the internal integrity of the test is high. The equality of the item means was tested using Hotelling’s T² test, and a significant difference was found between the means (Hotelling’s T² = 815.37, F = 34.465, *p* < 0.001). Hotelling’s T² test results show that the differences between the items are significant and the items contribute to the overall structure of the scale, supporting the structural integrity and measurement scope of the scale (Table 8).

**Table 6:** Reliability statistics of the scale

Scale Reliability Statistics				
	Mean	SD	Cronbach’s α	McDonald’s ω
ATOL Subdimension	2.040	0.684	0.876	0.885
ATODE Subdimension	2.560	0.881	0.930	0.930
TOTAL	2.290	0.712	0.938	0.940

*SD* Standard Deviation

**Table 7:** Reliability statistics of scale items

If item dropped					
	Mean	SD	Item-rest correlation	Cronbach’s α	McDonald’s ω
S1	3.18	1.28	0.554	0.937	0.938
S2	1.77	0.96	0.665	0.935	0.936
S3	1.50	0.82	0.588	0.936	0.937
S4	1.40	0.64	0.492	0.938	0.939
S5	1.96	1.07	0.653	0.935	0.936
S6	2.30	1.17	0.411	0.939	0.940
S7	1.89	0.94	0.423	0.938	0.940
S8	1.91	0.88	0.537	0.937	0.938
S9	2.21	0.98	0.576	0.936	0.938
S10	1.80	0.88	0.406	0.938	0.940
S11	2.39	1.25	0.731	0.934	0.935
S12	2.72	1.19	0.776	0.933	0.935
S13	2.79	1.20	0.700	0.934	0.936
S14	2.28	1.22	0.674	0.935	0.936
S15	2.87	1.21	0.694	0.934	0.936
S16	2.68	1.30	0.720	0.934	0.935
S17	2.92	1.26	0.737	0.933	0.935
S18	3.36	1.14	0.610	0.936	0.937
S19	2.94	1.18	0.696	0.934	0.936
S20	2.11	1.01	0.695	0.934	0.936
S21	2.08	1.07	0.749	0.933	0.935
S22	1.42	0.78	0.531	0.937	0.938

*SD* Standard Deviation

**Table 8:** Reliability statistics results

		*N* of Items	Value
Cronbach’s Alpha	Part 1	11	0.865
	Part 2	11	0.930
Correlation Between Forms		22	0.716
Spearman-Brown Coefficient	Equal Length	22	0.835
	Unequal Length	22	0.835
Guttman Split-Half Coefficient		22	0.814
Hotelling’s T² Test	T² Value		815.370
	F-Statistic Value		34.465
	*p*-value		< 0.001

In conclusion, the analyses show that the scale is highly valid and reliable. These results indicate that the scale can be adapted to dental students.

### Comparison of sociodemographic characteristics and scale results

In our study, No statistically significant difference was found between ATOL, ATODE subdimensions and total scale scores according to gender. ATOL subdimension was calculated as 23 ± 8 in males and 22 ± 7 in females and this difference was not statistically significant (*p* = 0.832). The ATODE subscale was 30 ± 11 in males and 28 ± 9 in females, and the difference was not statistically significant (*p* = 0.196). The total scale score was 52.98 ± 17.71 in males and 49.59 ± 14.85 in females and the difference between the groups was *p* = 0.204, which was not statistically significant.

In the comparisons of ATOL, ATODE subdimensions and total scale scores according to year of study, no significant difference was found in ATOL subdimension scores according to year of study (19 ± 6 for 1 st year, 23 ± 5 for 2nd year, 26 ± 11 for 3rd year, 21 ± 7 for 4th year and 23 ± 7 for 5th year, respectively) (*p* = 0.157). In the ATODE subdimension, a significant difference was found between the classes (23 ± 8 in the 1 st year, 23 ± 6 in the 2nd year, 32 ± 10 in the 3rd year, 28 ± 9 in the 4th year, and 30 ± 11 in the 5th year) (*p* = 0.008). As a result of post-hoc analyses, it was determined that the significant difference was between the 1 st year and the 3rd and 5th years, and between the 2nd year and the 3rd and 5th years. There was no statistically significant difference between year levels in terms of total scale score (42.56 ± 13.52 for 1 st year, 46.75 ± 9.84 for 2nd year, 55.3 ± 17.53 for 3rd year, 49.61 ± 15.46 for 4th year and 53.07 ± 16.38 for 5th year) (*p* = 0.082).

There was no significant relationship between age and ATOL subdimension (*r* = 0.113, *p* = 0.130). A positive and significant relationship was found between age and ATODE subscale (*r* = 0.193, *p* = 0.009). Similarly, a significant positive correlation was found between age and total scale score (*r* = 0.176, *p* = 0.018). Highly positive and statistically significant relationships were found between ATOL and ATODE (*r* = 0.719, *p* < 0.001), ATOL and total score (*r* = 0.888, *p* < 0.001) and ATODE and total score (*r* = 0.950, *p* < 0.001) (Table 9).

**Table 9:** Scale results comparison with sociodemographic data

			Gender						
			Male		Female				
			Mean ± std	Medyan (min-max)	Mean ± std	Medyan (min-max)	p		
ATOL Subdimension			23 ± 8	22 (11–55)	22 ± 7	22 (11–53)	0.832^Z^		
ATODE Subdimension			30 ± 11	29 (11–54)	28 ± 9	28 (11–52)	0.196^t^		
Scale Total Point			52.98 ± 17.71	53 (22–109)	49.59 ± 14.85	49.5 (22–98)	0.204^t^		
			Year of Study						
			1 st Year	2nd Year	3rd Year	4th Year	5th Year	p	post-hoc
ATOL Subdimension		Mean	19 ± 6; 20	23 ± 5; 23	26 ± 11; 23	21 ± 7; 21	23 ± 7; 24	0.157^Y^	
ATODE Subdimension		Mean	23 ± 8; 23	23 ± 6; 26	32 ± 10; 32	28 ± 9; 28	30 ± 11; 29	0.008^F^	1–3, 1–5, 2–3, 2–5
Scale Total Point		Mean	42.56 ± 13.52; 42.5	46.75 ± 9.84; 48	55.3 ± 17.53; 51.5	49.61 ± 15.46; 50	53.07 ± 16.38; 53.5	0.082^Y^	
Correlations									
			ATOL Subdimension	ATODE Subdimension	Scale Total Point				
Spearman's rho	Age	r	0.113	.193**	.176*				
		p	0.130	0.009	0.018				

Mann–Whitney U test; t: Independent samples t-test; Y: Kruskal–Wallis test; F: One-way ANOVA. Different superscript letters indicate statistically significant differences between groups (p < 0.05, Tukey’s post-hoc test). * p < 0.05, ** p < 0.01

As dental educators, researchers, and professionals, it is essential to review existing educational methods and even consider developing new systems to adapt to modern technology and effectively engage students. Exploring the impact of online learning on students will be valuable in formulating a new dental education framework. In our study, the online learning scale was administered to dental students at Atatürk University.

Only students from the Faculty of Dentistry at Atatürk University were invited to participate in this study, which stands out as a clear limitation of our research. All participating students volunteered, with most responses coming from fourth-year students, and the majority of participants were female. All participants were informed that the data collection process was entirely anonymous, helping to mitigate potential response bias.

In dental education, which inherently combines practical and theoretical learning, face-to-face classes are essential because skill-based clinical training and preclinical practical education remain challenging to transition entirely to online formats with current technology [36]. Although most students in our study acknowledged the benefits of online learning, many expressed concerns about being inadequately prepared for practical courses through online education, which aligns with findings from the existing literature [37–39]. Given the limitations of fully online education in dental training, it may be worthwhile to investigate the potential applicability of a blended learning model. However, further empirical research is needed to assess whether such a model can adequately integrate online and face-to-face components, especially in relation to students’ experiences with practical training.

Some studies have compared blended learning with fully online and face-to-face formats and reported that the blended model is equivalent to or, in some cases, superior to face-to-face education in terms of student grades [40–43]. Applications and artificial intelligence programs developed for online education could enhance the learning process. For instance, one study highlighted the need to explore a computer program as an additional teaching tool, which allowed students to learn topics, practice, learn from their mistakes, and develop problem-solving skills before clinical training with patients [44]. In line with these reports, the present study found that while students acknowledged the potential benefits of online modules and technology-assisted tools for reinforcing theoretical knowledge and problem-solving skills, they preferred these resources to be integrated into a blended model rather than replacing hands-on practical training. This preference underscores the importance of using such applications as preparatory and supplementary aids that complement, rather than substitute, face-to-face clinical education.

Our findings indicate that students prefer using online modules as a supplement to learning and do not support replacing traditional skill-based practical training with online instruction. These conclusions align with previous studies [45, 46] and suggest that students embrace online learning as a means to improve and enrich their education.

The time-management flexibility that online education offers to both students and educators is undeniable. Students’ responses to the scale support previous findings in dental education [14] suggesting that students may be inclined toward a blended learning model and that making course content and data available online could contribute positively to their education.

In the study, there was no significant difference between the Attitude Towards Online Learning (ATOL), Attitude Towards Online Dental Education (ATODE) and total scale scores according to gender, indicating that male and female students approached online education with similar attitudes. On the other hand, the presence of a significant difference in favour of upper class students in ATODE scores according to grade level suggests that attitudes towards online dental education developed more positively with the education process. The increase in ATODE and total scale scores with age also supports this situation. This may be related to differences in the ratio of theoretical to practical content across the dental curriculum. In Turkey, for example, a study reported that first-year students had significantly lower satisfaction scores, likely due to not having yet started clinical/practical training [20]. Similarly, at the University of Zagreb, clinical courses begin in the seventh semester, and the limitations of the online format in practical applications have been emphasised [47]. In South Korea, the same student cohort experienced a theoretical-heavy year in face-to-face format and a practice-heavy year online, which led to differences in satisfaction [48]. In Jordan, differences in adaptation to online learning between clinical and pre-clinical years have also been reported [21]. These findings suggest that the relationship between year level and attitudes observed in our study may be influenced by the proportion of theoretical and practical courses in the respective year’s curriculum.In addition, the high level of positive correlations between ATOL, ATODE and total scores indicate that the scale has a strong internal consistency.

The lower effort required for course participation and the high motivation for learning observed in online education could make it a valuable component of future dental curricula [39]. The results of this study overlap with previous studies evaluating attitudes towards online learning. Korkmaz [49] found a strong internal consistency and factor structure in the development of the Online Collaborative Learning Attitude Scale, as in our study. The E-learning Attitude Scale developed by Al-Musawi [50] also emphasised reasonable evidence of validity and reliability. All these studies cumulatively contribute to the argument supporting the psychometric validity of the scales developed in this study and underline their usefulness in the literature, particularly in dental education. These findings indicate that blended learning may become a future trend in dental education. However, further research is necessary to establish the efficiency and effectiveness of blended learning in dental training.

### Limitations

Our study has some limitations. The adaptation of the scale in a sample consisting only of dental students makes it difficult to generalise. The sample was selected intentionally, and representativeness is uncertain. The fact that it was not tested on students from different cultures and different disciplines also makes generalisation difficult. The study was conducted only at a single dental school, despite the existence of numerous faculties of dentistry in Turkey. In addition, the period during data collection were return to in-person classes might have influenced responses.

One limitation of the present study is that it assessed reliability only in terms of internal consistency. Test–retest reliability and responsiveness analyses were not conducted, which restricts the generalizability of the instrument’s temporal stability and sensitivity to change. Future studies should address these aspects to provide a more comprehensive evaluation of the instrument’s psychometric properties.

In addition to the above limitations, the present study did not evaluate all potential facets of psychometric validity. Specifically, convergent and discriminant validity were not tested through correlations with related and unrelated constructs, and measurement invariance across key subgroups (e.g., gender, year of study) was not assessed. Although differences in scores between genders and academic years were examined—partially reflecting a known-groups validity approach—future research should include dedicated analyses of convergent/discriminant validity and measurement invariance to provide a more comprehensive evaluation of the scale’s psychometric properties.

## Conclusion

The validity and reliability analyses conducted for the adaptation of the Online Learning Attitudes Scale to dental students demonstrated that the scale is valid and reliable. The item-total score correlation values ranged between 0.406 and 0.776, while the overall Cronbach’s alpha value was calculated as 0.938, and the subdimension Cronbach’s alpha values were found to be 0.876 and 0.930, respectively. Additionally, an examination of the fit indices revealed that χ²/df indicated good fit, RMSEA and SRMR indicated acceptable fit, and CFI indicated low fit. In addition, it was found that dental students’ attitudes towards online education developed positively depending on age and academic progress. Based on these findings, it has been shown that the “Medical Students’ Attitudes Towards Online Learning Scale” can be used to measure the attitudes of dental students towards distance/online learning.

### Future directions and scope

Studies conducted using our scale will increase generalisability. It will also reveal the attitude changes of dental students over time longitudinally.

## Supplementary Information

Supplementary Material 1.

## Data Availability

Kaşali, K. (2025). “Medical School Students’ Attitudes Towards Distance Education/Online Learning Scale” [Data set]. Zenodo. (10.5281/zenodo.14244844).
