# Supplementary material for: Validity and reliability study of the dental students of attitudes towards online learning scale
Source: BMC Med Educ. 2025 Dec 22;25:1704. doi: 10.1186/s12909-025-08296-z (PMC12723913; doi:10.1186/s12909-025-08296-z)
Supplement: Supplementary file 1 — Supplementary Material 1. [file 12909_2025_8296_MOESM1_ESM.docx]

**Tıp Fakültesi Öğrencilerinin Uzaktan Eğitim/Online Öğrenmeye Yönelik Tutumları**

Değerli Katılımcı;

Bu ölçme aracı uzaktan eğitim/online öğrenme ortamlarına yönelik tutumu ölçmek üzere hazırlanmıştır. Sizden beklenen ölçme aracında yer alan her bir ifadeyi okumanız ve bu ifadedeki durumunuzu yansıtan seçeneği işaretlemenizdir. Ölçme aracına adınızı yazmayınız. Hiçbir ifadeyi boş bırakmayınız. Katılımınız için teşekkürler.

| **Kesinlikle Katılmıyorum = 1; Katılmıyorum = 2; Kısmen Katılıyorum = 3; Katılıyorum =4; Kesinlikle Katılıyorum = 5** | | | | | | |
| --- | --- | --- | --- | --- | --- | --- |
|  | | **(1)** | **(2)** | **(3)** | **(4)** | **(5)** |
| **1** | Derslerle/stajlarla ilgili ders notlarına online erişim hekim yetiştirmede idealdir. | **(1)** | **(2)** | **(3)** | **(4)** | **(5)** |
| **2** | Uzaktan eğitim yöntemleriyle tıp eğitimi amacına ulaşabilir. | **(1)** | **(2)** | **(3)** | **(4)** | **(5)** |
| **3** | Uzaktan eğitimle hekim yetiştirilebilir. | **(1)** | **(2)** | **(3)** | **(4)** | **(5)** |
| **5** | Hekim yetiştirmek için gerekli beceriler online olarak kazandırabilir. | **(1)** | **(2)** | **(3)** | **(4)** | **(5)** |
| **6** | Bir hekimin sahip olması gereken tutumlar online olarak edinilebilir. | **(1)** | **(2)** | **(3)** | **(4)** | **(5)** |
| **7** | Hekimin, hastası ve yakınlarıyla nasıl iletişim kuracağı online olarak öğretilebilir. | **(1)** | **(2)** | **(3)** | **(4)** | **(5)** |
| **8** | Uzaktan eğitimle kazandığım bilgileri (örneğin tansiyon ölçme) hastayla karşılaştığımda beceri olarak sergileyebilirim. | **(1)** | **(2)** | **(3)** | **(4)** | **(5)** |
| **9** | Bir hastanın nasıl muayene edileceği online olarak öğretilebilir. | **(1)** | **(2)** | **(3)** | **(4)** | **(5)** |
| **10** | Zor haber verme (ölüm haberi verme gibi) becerisi online öğretilebilir. | **(1)** | **(2)** | **(3)** | **(4)** | **(5)** |
| **12** | Acil tıp uygulamaları online öğretilebilir. | **(1)** | **(2)** | **(3)** | **(4)** | **(5)** |
| **20** | Tıp eğitimindeki sınıf dersleri bir kayıp oluşturmadan online olarak verilebilir. | **(1)** | **(2)** | **(3)** | **(4)** | **(5)** |
| **21** | Sır  Sınıf derslerinin online sisteme geçirilmesi, ders çalışma programımı kişiselleştirmeme katkı sağlar. | **(1)** | **(2)** | **(3)** | **(4)** | **(5)** |
| **22** | Online eğitim görsel-işitsel ögelerin kullanımına katkı yapar. | **(1)** | **(2)** | **(3)** | **(4)** | **(5)** |
| **23** | Online eğitim fiziksel kısıtlılıklara sahip sınıf eğitiminden üstündür. | **(1)** | **(2)** | **(3)** | **(4)** | **(5)** |
| **25** | Online eğitime kolayca uyum sağlarım. | **(1)** | **(2)** | **(3)** | **(4)** | **(5)** |
| **26** | Online eğitim zamanını sınıf derslerinden daha verimli kullanmamı sağlar. | **(1)** | **(2)** | **(3)** | **(4)** | **(5)** |
| **27** | Uzaktan eğitim ortamındaki ders notlarından bilgi edinmekten hoşlanırım. | **(1)** | **(2)** | **(3)** | **(4)** | **(5)** |
| **28** | Dersler/stajlarla ilgili ders notlarına online erişim bana kendimi özgür hissettirir. | **(1)** | **(2)** | **(3)** | **(4)** | **(5)** |
| **31** | Online eğitim görsel, işitsel, etkileşimli olarak büyük zenginliktir. | **(1)** | **(2)** | **(3)** | **(4)** | **(5)** |
| **38** | Sınıf arkadaşlarımın online eğitim almaları için onları teşvik ederim. | **(1)** | **(2)** | **(3)** | **(4)** | **(5)** |
| **39** | Uzaktan eğitimi yaygınlaştıracak her tür çabayı desteklerim. | **(1)** | **(2)** | **(3)** | **(4)** | **(5)** |
| **40** | Sağlık bakanı olsam uzaktan eğitimle hekim yetiştirilmesini sağlarım. | **(1)** | **(2)** | **(3)** | **(4)** | **(5)** |

^22 madde ve iki alt ölçekten oluşan bir ölçme aracıdır. Ölçek beşli Likert tipi bir ölçümdür.Cronbach Alpha güvenilirlik değeri ölçeğin 0.96 olduğu bulunmuştur. Ölçek genelinden alınabilecek en yüksek puan:110, en düşük puan 22’dir. Ölçekte tersten kodlama/puanlama bulunmamaktadır. Alt ölçekler: çevrimiçi tıp eğitimine yönelik tutumlar (öğeler 1-11) ve Çevrimiçi öğrenmeye yönelik tutumlar (madde 12–22). Her boyut 11 maddeden oluşmakta olup, her bir boyuttan alınabilecek maksimum puan 55 minimum puan 11'dir.^ ^Kat puanı herkes kendisi belirleyebilir.^
